# Pre-clinical and clinical efficacy of curcumin as an anti-inflammatory agent for periodontitis. A systematic review

**DOI:** 10.21142/2523-2754-1204-2024-222

**Published:** 2024-11-23

**Authors:** Fiki Muhammad Ridho, Andika Julyanto Syachputra, Anisa Dias Nur’aini, Kamailiya Ulfah, Muhamad Faqih, Andang Nurhuda

**Affiliations:** 1 Dental Profession Program, Faculty of Dental Medicine, Universitas Airlangga. Surabaya, Indonesia. fikimuhammadridho@gmail.com Dental Profession Program Faculty of Dental Medicine Universitas Airlangga Surabaya Indonesia fikimuhammadridho@gmail.com; 2 Department of Biology, Faculty of Biology, Universitas Gadjah Mada. Yogyakarta, Indonesia. andikajulyant07@gmail.com Department of Biology Faculty of Biology Universitas Gadjah Mada Yogyakarta Indonesia andikajulyant07@gmail.com; 3 Pharmacist Profession Program, Faculty of Pharmacy, Universitas Ahmad Dahlan. Yogyakarta, Indonesia. ddiasanisa@gmail.com Pharmacist Profession Program Faculty of Pharmacy Universitas Ahmad Dahlan Yogyakarta Indonesia ddiasanisa@gmail.com; 4 Veterinarian Profession Program, Faculty of Veterinary Medicine, Universitas Airlangga. Surabaya, Indonesia. kamailiyaulfah@gmail.com Veterinarian Profession Program Faculty of Veterinary Medicine Universitas Airlangga Surabaya Indonesia kamailiyaulfah@gmail.com; 5 Department of Bioprocess Engineering, Faculty of Chemical and Energy Engineering, Universiti Teknologi Malaysia. Johor Bahru, Malaysia. faqih-20@graduate.utm.my Department of Bioprocess Engineering Faculty of Chemical and Energy Engineering Universiti Teknologi Malaysia Johor Bahru Malaysia faqih-20@graduate.utm.my; 6 Undergraduate Program, Faculty of Mathematics and Natural Sciences, Universitas Negeri Surabaya. Surabaya, Indonesia. andang.19010@mhs.unesa.ac.id Undergraduate Program Faculty of Mathematics and Natural Sciences Universitas Negeri Surabaya Surabaya Indonesia andang.19010@mhs.unesa.ac.id

**Keywords:** anti-inflammatory, curcumin, periodontal disease, periodontitis, antiinflamatorio, curcumina, enfermedad periodontal, periodontitis

## Abstract

**Introduction:**

There is ongoing exploration into herbal treatments to identify adjunct therapies with minimal side effects. One such treatment involves curcumin from turmeric (*Curcuma longa*). This study aims to review the efficacy of curcumin as an anti-inflammatory agent for periodontitis along with the mechanisms of action involved.

**Methods:**

A systematic review of pre-clinical and clinical studies published on Scopus, PubMed, ScienceDirect, and Google Scholar up to May 2024 was employed following the PRISMA guidelines. Three tools were used for risk of bias assessment, namely the QUIN tool for *in vitro* studies, the SYRCLE’s RoB for *in vivo* studies, and the Cochrane RoB 2 for RCTs. Finally, nineteen studies were included for review.

**Results:**

This study highlights curcumin’s efficacy in addressing periodontitis through diverse mechanisms. Curcumin demonstrated efficacy in attenuating inflammation within periodontal tissue by inhibiting several pro-inflammatory cytokines and mediators such as interleukin (IL)-1, IL-6, tumor necrosis factor (TNF)-α, matrix metalloproteinases (MMPs), prostaglandin E2 (PGE2), cyclooxygenase (COX)-2, while concurrently increasing IL-4 and IL-10. In addition, several transcription factors such as nuclear factor-kappa B (NF-κB) and signal transducer and activator of transcription 1 (STAT1) were also inhibited by curcumin. Administration of curcumin has additionally been demonstrated to reduce other biomarkers of periodontitis, including C-reactive protein (CRP), alkaline phosphatase (ALP), and procalcitonin (PCT).

**Conclusion:**

Curcumin has been shown to be effective as an adjunct therapeutic agent for periodontitis due to its anti-inflammatory effects by reducing the inflammatory response through a diverse range of mechanisms of action.

## RESUMEN

**Introducción:**

Actualmente, se están realizando estudios sobre tratamientos a base de hierbas para identificar terapias complementarias con efectos secundarios mínimos. Uno de estos tratamientos incluye la curcumina de la cúrcuma (*Curcuma longa*). El presente estudio tiene como objetivo revisar la eficacia de la curcumina como agente antiinflamatorio para la periodontitis junto con los mecanismos de acción involucrados.

**Métodos:**

Se empleó una revisión sistemática de estudios preclínicos y clínicos publicados en Scopus, PubMed, ScienceDirect y Google Scholar, hasta mayo de 2024, siguiendo las pautas PRISMA. Se utilizaron tres herramientas para evaluar el riesgo de sesgo: QUIN *tool* para estudios *in vitro*, SYRCLE’s RoB para estudios *in vivo* y Cochrane RoB 2 para RCT. Finalmente, se incluyeron diecinueve estudios para su revisión.

**Resultados:**

Este estudio destaca la eficacia de la curcumina en el tratamiento de la periodontitis a través de diversos mecanismos. La curcumina demostró eficacia para atenuar la inflamación en el tejido periodontal al inhibir varias citocinas y mediadores proinflamatorios como interleuquinas (IL)-1, IL-6, factor de necrosis tumoral (TNF)-α, metaloproteinasas de la matriz (MMP), prostaglandina E2 (PGE2), ciclooxigenasa (COX)-2, mientras que, simultáneamente, aumenta IL-4 e IL-10. Además, varios factores de transcripción, como factor nuclear kappa B (NF-κB) y transductor de señal y activador de la transcripción 1 (STAT1), también fueron inhibidos por la curcumina. También se ha demostrado que la administración de curcumina reduce otros biomarcadores de periodontitis, incluidos proteína C reactiva (CRP), fosfatasa alcalina (ALP) y procalcitonina (PCT).

**Conclusiones:**

Se ha demostrado que la curcumina es eficaz como agente terapéutico complementario para la periodontitis, debido a sus efectos que reducen la respuesta inflamatoria a través de una amplia gama de mecanismos de acción.

## INTRODUCTION

Periodontitis is an inflammatory condition on the tooth-supporting tissue caused by specific microorganisms, leading to the progressive periodontal ligament and alveolar bone destruction ^1^. Erythematous, swelling, bleeding on probing, periodontal pocket development, clinical attachment loss, loose teeth, tooth migration, gingival margin recession, and radiographic alveolar bone loss are some of the clinical indicators of periodontitis ^2^. According to a meta-analysis of epidemiological data, 62% of population have periodontitis between 2011 and 2020, with severe periodontitis affecting as much as 24% of the population, where the prevalence data experienced a significant increase compared to the 1990-2010 period ^3^. Furthermore, various evidence reported that periodontitis significantly influences and is linked to systemic diseases, including cardiovascular disease ^4^, diabetes ^5^, metabolic syndrome ^6^, cancers ^7^^-^^10^, pregnancy complications ^11^^,^^12^, renal disease ^13^, sexual dysfunction ^14^^,^^15^, and other systemic diseases, making it a global health concern.

Periodontitis is classified as a complex type of chronic inflammatory disease. Pathogens release lipopolysaccharides that cause damage to periodontal tissues, leading to an inflammatory response characterized by increased production of pro-inflammatory cytokines, prostaglandins (PGs), and matrix metalloproteinases (MMPs) ^16^. Chronic inflammation in periodontitis plays a major role in the destruction of periodontal tissues and loss of alveolar bone. Therefore, control of excessive inflammation in periodontitis is believed to drastically reduce to no periodontal bone loss ^17^.

Currently, mechanical treatment, including scaling and root planning (SRP), sub- and supragingival irrigation, administration of local and systemic antibiotics, and chemotherapy are the main modalities for treating periodontitis ^18^^,^^19^. While mechanical treatment remains the most effective technique, the use of chemical mouthwashes is also essential for plaque control, prevention of biofilm formation, and the initial phase of periodontal therapy. Chlorhexidine is the most effective chemotherapy agent for treating periodontitis, yet studies have shown that this agent has adverse effects such as stains or spots on the teeth, changes in taste, oral numbness, oral pain, xerostomia, tongue discoloration, hypogeusia, and burning mouth syndrome ^20^. Moreover, the administration of local and systemic antibiotics for periodontitis is linked to adverse effects, including allergies, nephritis, and disorders of the hematology, digestive, and nervous systems, as well as contributes to increased antibiotic resistance within the oral microflora ^21^^,^^22^. In relation to inflammation in periodontitis, in addition to being considered to be able to reduce the rate of alveolar bone loss, nonsteroidal anti-inflammatory drugs (NSAIDs) also show adverse effects on the gastrointestinal, cardiovascular, kidneys, as well as hepatotoxicity and dermatological reactions ^23^.

Community interest in traditional treatment using herbal medicine continues to grow. Turmeric (*Curcuma longa*) attains huge attention due to its curing ability with insignificant side effects. Curcumin (diferuloylmethane) is one of the most abundant compounds found in turmeric ^24^. In dentistry, curcumin is effective in treating numerous oral disorders, such as oral cancer, periodontal disease, oral lichen planus, dental caries, oral mucositis, and other oral mucosal diseases ^25^^-^^29^. For instance, curcumin shows positive effects in treating periodontitis because it has anti-inflammatory effects against gingival inflammation and antibacterial effects against periodontopathogens ^30^^,^^31^. A study has shown that curcumin has been shown to provide anti-inflammatory effects through the regulation of various inflammatory mediators and inflammatory signaling pathways ^32^.

Despite growing research on the effects of curcumin, there is a noticeable gap in comprehensive reviews that systematically explore its specific anti-inflammatory effects and mechanisms in the context of periodontitis. Given the increasing prevalence of periodontitis, it is crucial to prevent and reverse periodontal tissue damage and alveolar bone loss due to periodontitis-related inflammation. Additionally, the shortcomings of conventional periodontal treatments highlight the need for alternative therapies. This systematic review aims to address this gap by summarizing current evidence on the anti-inflammatory properties of curcumin and its underlying mechanisms in the treatment of periodontitis. This contribution is critical in advancing our understanding of alternative treatments that can improve patient outcomes.

## MATERIAL AND METHODS

### Review methodology

This study is a systematic review of preclinical and clinical studies following the PRISMA guidelines, with the research question being "what is the anti-inflammatory effect of curcumin on periodontitis and what is the underlying mechanism of action?". The population, intervention, comparison, and outcomes (PICO) framework was utilized to address the research question, shown in Table 1.

**Table 1:** PICO framework.

Element	Details
Population	Cells, animal models, or humans with periodontitis
Intervention	Curcumin from *Curcuma longa* or *Curcuma* spp.
Comparison	Subjects with placebo control, standard periodontal treatments, or no treatment
Outcomes	Anti-inflammatory effects and its underlying mechanism of action.

### Information sources and search terms

Several electronic databases, including Scopus, PubMed, ScienceDirect, and Google Scholar, were used in this study. Two authors (F.M.R. and A.J.S.) performed the first literature search in October 2023, then a re-search was conducted in May 2024 to avoid missing articles published after the first search. We searched for articles in predetermined databases using the following keywords: (inflammation OR inflammatory OR anti-inflammation OR anti-inflammatory) AND (curcumin OR curcuma OR turmeric OR diferuloylmethane) AND (periodontitis OR periodontal OR periodontal disease).

### Eligibility criteria

All articles obtained from the literature search were then screened based on eligibility criteria by two authors (A.D.N. and K.U.). The inclusion criteria applied included original research in the form of preclinical or clinical research, research subjects which are cells or animal models induced by periodontitis, or patients with periodontitis, articles written in English, peer-reviewed, and fully accessible. On the other hand, we excluded all review articles, short communications, and editorials, as well as studies with combined interventions with other agents or curcumin derivatives. The study included all articles published until May 2024, without any restrictions on the publication year.

## Risk of bias assessment

The risk of bias assessment was conducted using three different tools according to the type of study. The Quality Assessment Tool for In Vitro Studies (QUIN) tool was used to evaluate in vitro studies with a score of 2 given for adequately specified, 1 for inadequately specified, and 0 for not specified. A study is rated low risk of bias, medium risk of bias, and high risk of bias if the final score is >70%, 50-70%, and <50%, respectively ^33^. The Systematic Review Centre for Laboratory Animal Experimentation (SYRCLE's RoB) tool was utilized for in vivo studies with "Yes", "No", and "Unclear" indicating a low, high, and unclear risk of bias, respectively ^34^. Finally, the Cochrane Risk of Bias 2 (RoB 2) tool was used to assess RCT with a judgement of "low risk of bias", "some concerns", or "high risk of bias" ^35^. The risk of bias evaluation was carried out by two reviewers (F.M.R. and A.D.N.) and validated by one author (A.J.S.). All differences of opinion during risk of bias assessment were resolved through in-depth discussions and careful decision-making with the other authors.

### Data extraction

The final stage is data extraction, conducted by two authors (K.U. and M.F.), where tables are used to summarize all the important information, such as author, study design, subjects, intervention with curcumin along with the dose given, control applied, and results in the form of mechanism of action involved. Finally, we conducted a qualitative analysis of the data obtained and summarized all data and mechanism of action of curcumin on periodontitis.

## RESULTS

### Study selection

**Figure 1:**
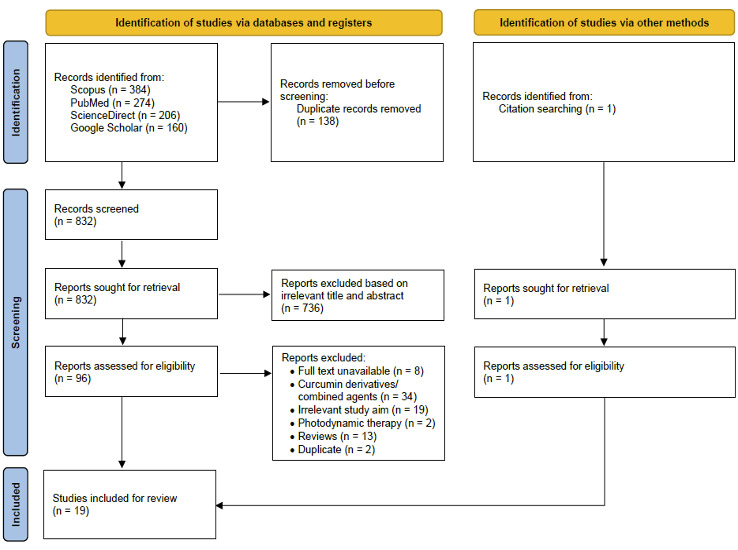
Study selection process.

A total of 1,024 raw data were obtained from database searches. 138 duplicate articles were eliminated with the use of Mendeley reference manager. Subsequently, the remaining 832 articles were screened based on title and abstract, resulting in 736 reports being excluded due to irrelevance. The screening results left 96 articles which were then assessed for eligibility. At this stage, a total of 78 articles were eliminated, 18 articles remain. We also searched using other methods, namely citation searches, and found 1 article which was then added. Finally, 19 articles were included for review, consisting of 5 *in vitro* studies, 10 *in vivo* studies, 1 *in vitro* and *in vivo* study, and 3 randomized-controlled studies (RCTs). The entire study selection process is depicted in Figure 1.

### Characteristics of the included studies

All summary results that we extracted from nineteen included research are presented in Table 2.

**Table 2:** Summary of the included articles.

Author	Study Design	Subjects	Dose and Duration of Treatment	Control	Results
Chen et al. ^36^	*In vitro*	Macrophage RAW264.7 cells	5-30 µmol/L (24 hours)	Untreated	Significantly reduced TNF-α and IL-1β synthesis. Suppressed LPS-induced NF-κB activity.
Guimarães et al. ^37^	*In vivo*	60 Holtzman rats	30 and 100 mg/kg (15 days)	Vehicle	Suppressed inflammatory cells. Inhibited NF-κB activation. Inhibited PGE2 mRNA synthesis.
Kim ^38^	*In vitro*	Macrophage RAW 264.7 cells	1-20 µM (24 hours)	Untreated	Inhibited IL-6 and TNF-α expression. Inhibited IL-6 production at a dose of 20 µM up to 83% inhibition. Inhibited the nuclear translocation of the NF-κB p50 subunit, which blocked NF-κB signaling. Attenuated NF-κB p65 and p50 subunit DNA binding activity. Inhibited STAT1 phosphorylation.
Hu et al. ^39^	*In vitro*	Human gingival fibroblasts (HGFs) cells	5-30 µmol/L (24 hours)	Untreated	Significantly attenuated COX-2 expression. Reduced COX-2 mRNA levels. Significantly inhibited NF-κB activity.
Zhou et al. ^40^	*In vivo*	30 Wistar rats	100 mg/kg (30 days)	I: Saline II: Vehicle	Decreased IL-6 and TNF-α.
Hosadurga et al. ^41^	*In vivo*	21 Wistar rats	2% curcumin gel (6 days)	I: Untreated II: Plain gel	Inhibited edema by approximately 43%. Decreased GI scores.
Corrêa et al. ^42^	*In vivo*	40 Wistar rats	100 mg/kg (30 days)	Placebo	Insignificantly decreased IL-1β concentration. Significantly increased IL-4 concentration.
Guru et al. ^43^	*In vitro*	Gingival tissue samples obtained from 30 patients with generalized moderate-to-severe chronic periodontitis, aged >35 years	300, 500, 1000, and 1,500 µg/mL (1 hour)	Doxycycline	Significantly inhibited MMP-9 activity.
Curylofo-Zotti et al. ^44^	*In vivo*	50 LPS-injected Holtzman rats	100 mg/kg (15 days)	No treatment	Reduced infiltration of PMN cells and mononuclear cells.
Guimaraes-Stabili et al. ^45^	*In vivo*	60 Holtzman rats	300 mg/kg (5 and 15 days)	I: Vehicle II: Untreated	Significantly inhibited NF-κB activity.
Xiao et al. ^46^	*In vitro*	Normal Wistar rats’ gingival tissue	10 and 20 µM (24 hours)	Untreated	Significantly reduced TNF-α and IL-1β levels. Significantly inhibited activation of NF-κB produced by LPS.
	*In vivo*	24 Wistar rats	30 and 100 mg/kg (Not reported)	None	Reduced the level of gingival inflammation.
Zambrano et al. ^47^	*In vivo*	16 Holtzman rats	3 µL of nano-curcumin injected twice a week (28 days)	Empty nanoparticles	Significantly reduced the number of PMN cells and mononuclear cells. Inhibited the activation of p38 MAPK and NF-κB.
Mirza et al. ^48^	RCT	60 patients with chronic periodontitis, aged 30-44 years	10 mL solution of 1% curcumin, irrigated sub-gingivally (3 and 7 days after SRP)	I: Chlorhexidine II: SRP only	Significantly reduced serum ALP levels. Decreased CRP concentrations.
Toraya et al. ^49^	*In vitro*	HGEPs cells cultured with P. gingivalis	0.01-100 µM, every 3 days (18 days)	Untreated	Significantly downregulated the expression of IL-6, IL-1β, TNF-α, TIMP-1, and MMP-9. Inhibited NF-κB activation.
Pérez-Pacheco et al. ^50^	RCT	20 patients with periodontitis, aged 37-62 years	50 µg of nano-curcumin (Once, given after SRP)	Empty nanoparticles	Reduced IL-6 levels. Inhibited the increase in IL-1α levels, as in the control group. Reduced TNF-α.
Yetkin Ay et al. ^51^	*In vivo*	35 Wistar rats	30 mg/kg (15 days)	DMSO	Decreased IL-1β expression. Decreased IL-6 expression.
Abdel-Fatah et al. ^52^	RCT	54 patients with stage II grade A periodontitis, aged 30-55 years	2% curcumin gel given once after SRP (42 days)	I: Healthy controls II: SRP only	Significantly reduced salivary PCT levels, but not significantly compared to positive controls.
Mohammad et al. ^53^	*In vivo*	50 Wistar rats	12.5 µg/mL gel containing 95% of curcumin, given after SRP (7 days)	I: Healthy rats II: No treatment III: Tetracycline IV: SRP	Significantly decreased the concentration of IL-6, MMP-8, CRP, and ALP. Insignificantly increased the level of IL-10.
Krismariono & Purnama S ^54^	*In vivo*	48 Wistar rats	1% curcumin given every 3 days (14 days)	Untreated	Inhibited MMP-7 activity.

### Risk of bias

**Figure 2:**
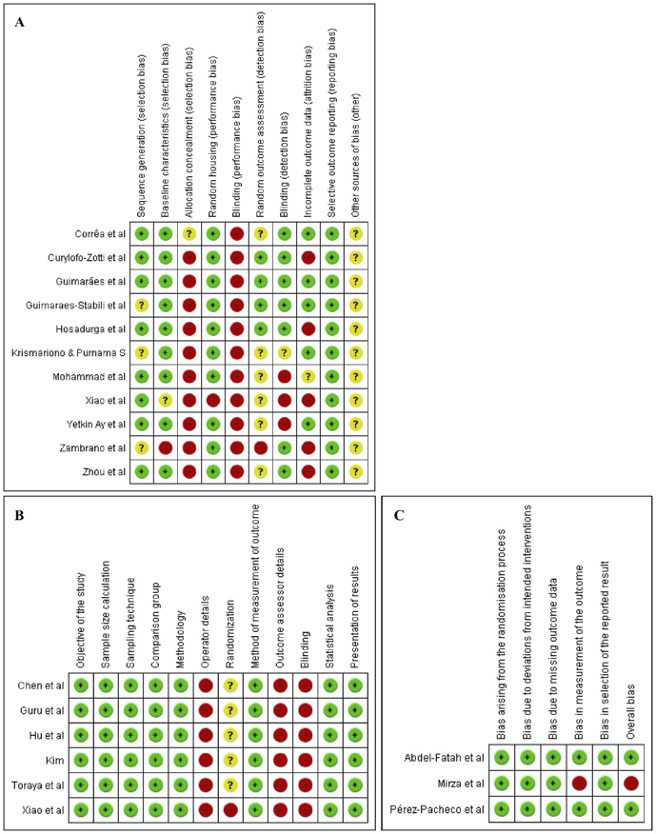
Risk of bias summary for *in vivo* studies using the SYRCLE’s RoB (A), for *in vitro* studies using the QUIN tool (B), and for RCTs using RoB 2 (C).

Risk of bias assessment was performed on all included studies. One study ^46^ which is an *in vitro* and *in vivo* study was assessed using the QUIN tool for the in vitro part and the SYRCLE’s RoB for the *in vivo* part. In the assessment of *in vitro* studies using the QUIN tool, the assessment results showed that five studies were rated as low risk of bias with an overall score of 71% and one study was rated as medium risk of bias with an overall score of 67%. In the assessment of *in vivo* studies using the SYRCLE's RoB, it was shown that the majority of studies showed a high risk of bias in allocation concealment (selection bias) and blinding (performance bias), as well as an unclear risk of bias for other sources of bias due to the lack of clarity regarding other biases. In the assessment of RCTs using RoB 2, we highlighted two studies as having low risk of bias and one other as having high risk of bias. The entire risk of bias summary is clearly depicted in Figure 2.

## DISCUSSION

The complex inflammatory nature of periodontitis involves multiple immune responses, making it imperative to explore alternative therapeutic agents, such as curcumin, known for its potential anti-inflammatory effects. The present study provides an update on the current evidence regarding the anti-inflammatory effects of curcumin on periodontitis and its mechanism of action with a comprehensive review.

The inflammatory condition that characterizes periodontitis is the body’s self-defense mechanism, involving cells and mediators from the innate and adaptive immune systems ^55^. Both acute and chronic inflammation are strongly influenced by a number of pro-inflammatory cytokines, such as interleukin (IL)-1α, IL-1β, IL-6, IL-17, tumor necrosis factor (TNF)-α; as well as IL-10 that has the opposite role ^56^. In addition, inflammatory conditions in periodontitis also lead to elevated levels of IL-1β and MMP-8 ^57^. We emphasize that natural curcumin is effective in ameliorating inflammatory conditions in periodontitis through diverse mechanisms, based on the review of all included studies ^36^^-^^54^. Additionally, Hosadurga and colleagues ^41^ found that curcumin inhibited edema by up to 43% and reduced GI scores, whereas the control group experienced an increase in GI scores indicating ongoing inflammation. Furthermore, study conducted by Guru and colleagues ^43^ revealed that curcumin is more effective as an anti-inflammatory compared to doxycycline, where doxycycline is considered effective as an adjunct to SRP because of its ability to anti-inflammatory ^58^.

Of all the included articles, one *in vitro* study looked into the phosphorylation of signal transducer and activator of transcription 1 (STAT1). The study found that curcumin effectively inhibits STAT1 phosphorylation ^38^. An essential function of STAT1 is to mediate the actions of several cytokines, including converting interferon (IFN)-γ signals into the expression of genes such as cyclooxygenase (COX), and to play a significant role in regulating various disorders related to inflammation and immunity ^59^^-^^61^. Other evidence suggests that significantly increased levels of STAT1 are found in individuals with chronic and aggressive periodontitis ^62^, and that STAT1 activation then promotes IL-1β and TNF-α ^63^. Therefore, inhibition of STAT1 phosphorylation by curcumin contributes to the improvement of periodontitis conditions by inhibiting several cytokines, resulting in a reduction in inflammatory conditions in periodontal tissue.

Several findings in this study demonstrate that curcumin strongly inhibits the rise in IL-1α levels ^50^ and reduces IL-1β levels ^36^^,^^42^^,^^46^^,^^49^^,^^51^. In periodontitis, IL-1 contributes to various processes required in the inflammatory response by increasing the synthesis of adhesion molecules, facilitating leukocyte migration, activating T and B lymphocytes, triggering inflammatory mediators and metalloproteinases production, stimulating alveolar bone resorption, and inhibiting tissue regeneration ^64^. Two of the IL-1 members are IL-1α and IL-1β. IL-1α initiates and maintains the inflammatory process in periodontal tissue ^65^, while IL-1β functions in responding to other inflammatory cytokines, notably TNF-α, which is the main mediator of periodontal inflammation and bone destruction ^66^, as well as increasing the regulation of MMP-9 expression and PGE2 synthesis in fibroblast cells ^65^^,^^67^. Surprisingly, several papers found curcumin significantly reduced TNF-α expression ^36^^,^^37^^,^^40^^,^^46^^,^^49^^,^^50^, where TNF-α is the pro-inflammatory cytokine that contributes to periodontitis-related bone resorption, and correlates with PGE2 biosynthesis ^68^^,^^69^. It is possible to draw the conclusion that curcumin reduces the concentrations of IL-1α and IL-1β, apart from reducing the inflammatory response in periodontal tissue, also contributes to reducing TNF-α expression.

A number of anti-inflammatory cytokines, notably IL-4, IL-10, and IL-13, contribute significantly to the inhibition of COX-2 induction ^70^. We highlight that curcumin significantly increases IL-4 concentrations ^42^ and insignificantly IL-10 levels ^53^ in subjects with periodontitis. Increasing IL-4 has an impact on inhibiting COX-2 activity and COX-2 mRNA ^71^. Meanwhile, IL-10 participates in suppressing and limiting the inflammatory response ^72^^,^^73^. Furthermore, evidence revealed that IL-10 is also regulated by one of the main pathways, namely mitogen-activated protein kinase (MAPK), where the p38 MAPK and extracellular signal-regulated kinase (ERK) pathways also regulate IL-10 production ^74^^,^^75^. Interestingly, one in vivo study in mice with periodontitis suggested that curcumin inhibits p38 MAPK activation ^47^. Evidence supports these findings that curcumin effectively inhibits several inflammation-related signaling including MAPK ^76^. In conclusion, inhibition of p38 MAPK activation by curcumin promotes an increase in IL-10 levels. In addition, curcumin also effectively increases IL-4 and IL-10 levels, which further contributes to COX-2 inhibition.

Curcumin effectively attenuates COX-2 expression and reduces COX-2 mRNA levels ^39^. Generally, COX-2 is expressed in periodontal tissue undergoing an inflammatory process, and play a role in arachidonic acid to PG conversion ^77^^,^^78^. COX-2 is essential in the synthesis of PGE2 and is engaged in the inflammatory process in periodontitis ^79^. In addition, periopathogens are also believed to stimulate monocytes and fibroblasts to produce IL-1α, IL-1β, and TNF-α, which in turn promotes COX-2 ^80^. Interestingly, curcumin also significantly inhibits PGE2 synthesis in periodontitis ^37^. PGE2 is known to have pro-inflammatory effects mediated by various receptors, such as a vasodilator that causes swelling, increases pain responses through sensory nerve stimulation, and encourages T helper 17 (TH17) cell activation which is typified by the release of IL-17 ^81^. Thus, inhibition of COX-2 by curcumin contributes to a reduction in PGE2 synthesis, a strong inducer of bone resorption. Inhibition of COX-2 and PGE2 by curcumin indicates a positive anti-inflammatory effect.

In addition to IL-6, two potent cytokines that activate nuclear factor-kappa B (NF-κB) are IL-1β and TNF-α ^82^. NF-κB, a transcription factor, plays a vital and complex function in regulating a large number of genes related to various inflammatory and immune response processes ^83^^,^^84^. Effectively, the inflammatory process in periodontitis was inhibited and reduced by curcumin through inhibition of NF-κB activity, as evidenced by the results of the eight reports ^36^^-^^39^^,^^45^^-^^47^^,^^49^. We previously mentioned that NF-κB activators, namely IL-1β and TNF-α, were decreased by curcumin administration. Interestingly, curcumin also decreases IL-6 expression in periodontitis ^37^^,^^38^^,^^40^^,^^49^^-^^51^^,^^53^. In addition to inhibiting NF-κB activity, effective prevention and therapy of various chronic inflammatory diseases are facilitated by IL-6 suppression ^85^. Furthermore, it has been demonstrated that blocking the NF-κB signaling pathway lowers inflammation and is linked to a drop in pro-inflammatory cytokine expression ^86^. As a result, curcumin’s inhibition of NF-κB activity suggests that it has an anti-inflammatory effect on periodontitis, resulting in tissue inflammation in the tissue.

Another indicator of the inflammatory phase that occurs in periodontitis is high accumulation of polymorphonuclear (PMN) cells. In inflammation that occurs in the oral cavity, PMN cells respond by leaving the blood circulation, then infiltrating the periodontal tissue ^87^. Two studies agreed that the number/infiltration of PMN and mononuclear cells were shown to have decreased after curcumin administration ^44^^,^^47^. Degranulation and infiltration of PMNs leads to MMPs release when pro-inflammatory mediators are activated ^88^^-^^90^, whereas, furthermore, PMN cells contribute to the production of MMP-9 and MMP-8 enzymes, leading to the destructive and inflammatory phase of periodontal disease ^91^. These findings are associated with several lines of evidence showing that curcumin effectively inhibits MMP-9 activity ^43^^,^^49^, MMP-8 ^53^, and MMP-7 ^54^. MMP-9 and MMP-8 are valid disease indicators and as regulators of inflammation, proven to be increased in patients with periodontal disease ^92^^,^^93^, as well as an increase in tissue inhibitor of metalloproteinase (TIMP)-1 in saliva ^94^. TIMPs are also recognized to be crucial in regulating MMPs activity, one of which is TIMP-1 ^95^. Additionally, MMP-3 and MMP-7 levels also increase in gingival crevicular fluid (GCF) in individuals with periodontal disease ^96^. In this context, salivary TIMP-1 is also significantly inhibited by curcumin ^49^. Evidence concludes that an imbalance between TIMPs and MMPs contributes to the pathological process ^97^; consequently, the capacity of curcumin to reduce MMP and TIMP levels while reducing the number of PMN cells suggests that the compound has a synergistic anti-inflammatory impact.

In addition to the previously mentioned, biomarkers-substances that can predict disease conditions in patients-show great promise for use as markers for detecting and monitoring the periodontitis progression ^98^. Several studies found that C-reactive protein (CRP), alkaline phosphatase (ALP), and procalcitonin (PCT) are three of various inflammatory biomarkers in periodontitis that are often used to detect and monitor disease progression ^99^^-^^103^. In this instance, given the substantial reduction in serum ALP ^48^^,^^53^, CRP ^48^^,^^53^, and salivary PCT levels ^52^, curcumin is considered effective in periodontitis therapy. The study found that periodontal treatment in patients with periodontitis had lower peak serum levels of inflammatory markers than those who were not treated ^104^. The success of periodontal treatment was also seen when administering curcumin as adjunct therapy, as evidenced by a reduction in several biomarkers such as ALP, CRP, and PCT.

The present review demonstrates that curcumin exhibits significant anti-inflammatory effects in periodontitis, primarily through the modulation of inflammatory cytokines. Additionally, curcumin effectively reduces the activity of MMPs and several transcription factors, which play a crucial role in periodontal inflammation. These findings highlight curcumin's potential as an adjunctive therapy to improve the outcomes of conventional periodontal treatments.

## CONCLUSIONS

Curcumin is the dominant polyphenol found in turmeric and has a healing effect on several disease conditions, one of which is used for periodontitis because it has been proven to have anti-inflammatory effects. This is evidenced by inhibiting or decreasing pro-inflammatory cytokines and mediators as well as increasing anti-inflammatory cytokines and mediators, and disrupting inflammation-related factors. This study highlights that curcumin is advantageous in inhibiting pro-inflammatory cytokines and mediators, including TNF-α, MMPs, IL-1, IL-6, PGE2, COX-2, while concurrently increasing anti-inflammatory cytokines and mediators, such as IL-4 and IL-10. Furthermore, several transcription factors, including NF-κB and STAT1, were also found to be inhibited by curcumin. Its administration has also been associated with reduced levels of other biomarkers of periodontitis, including CRP, ALP, and PCT.

Although this study provides evidence of anti-inflammatory effect of curcumin on periodontitis, larger and more diverse clinical trials and robust methods are required to establish the efficacy and safety of curcumin in a larger population, including RCT with larger samples and longer duration of observation to confirm these findings. Future research regarding curcumin dosage and formulations, including nanoforms and combinations with other therapeutic agents, may also be warranted to determine which is most effective in reducing periodontal inflammation. In addition, future research in special populations such as people with diabetes mellitus, smokers, and elderly groups to assess the safety and effectiveness of curcumin in these population is strongly needed.

## DECLARATIONS

Ethics approval: Not applicable.

Availability of data and material: This article contains all supplementary materials. All further information that support the study’s findings is available upon reasonable request from the corresponding author.
